# Lessons Learned from the Transgenic Huntington's Disease Rats

**DOI:** 10.1155/2012/682712

**Published:** 2012-07-18

**Authors:** Rinske Vlamings, Dagmar H. Zeef, Marcus L. F. Janssen, Mayke Oosterloo, Frederic Schaper, Ali Jahanshahi, Yasin Temel

**Affiliations:** ^1^Department of Neuroscience, Maastricht University Medical Center, 6229 ER Maastricht, The Netherlands; ^2^Department of Neurology, Maastricht University Medical Center, 6202 AZ Maastricht, The Netherlands; ^3^Department of Neurosurgery, Maastricht University Medical Center, 6202 AZ Maastricht, The Netherlands

## Abstract

Huntington's disease (HD) is a fatal inherited disorder leading to selective neurodegeneration and neuropsychiatric symptoms. Currently, there is no treatment to slow down or to stop the disease. There is also no therapy to effectively reduce the symptoms. In the investigation of novel therapies, different animal models of Huntington's disease, varying from insects to nonhuman primates, have been created and used. Few years ago, the first transgenic rat model of HD, carrying a truncated huntingtin cDNA fragment with 51 CAG repeats under control of the native rat *huntingtin* promoter, was introduced. We have been using this animal model in our research and review here our experience with the behavioural, neurophysiological, and histopathological phenotype of the transgenic Huntington's disease rats with relevant literature.

## 1. Introduction

Huntington's disease (HD) is an autosomal dominant neurodegenerative disorder ultimately leading to death, approximately 15–20 years after the first symptoms appear. The prevalence of HD in Europe and both American continents is reported to be approximately 5–7 in 100,000 and affects both sexes with the same frequency [1]. The disease is characterized by abnormal movement (chorea and hypokinesia), cognitive impairments, and psychiatric disturbances, which are progressive. These symptoms substantially limit social and professional functioning. Treatments to prevent death, slow down progression, or delay the onset of HD are still lacking [2].

HD is caused by a trinucleotide repeat expansion, an increase in the number of CAG repeats, in the *HD* gene, (*IT15*) [3]. There is an inverse correlation between the number of repeats and the age of onset in HD [4]. The number of repeats is negatively correlated with the age of onset and positively with disease progression [5]. The *HD* gene was the first autosomal disease locus to be mapped by genetic linkage analysis in 1983 [6] and is located on the short arm of chromosome 4. The *HD* gene encodes polyglutamine repeats in the *huntingtin* (*HTT*) protein [7], which results in a stretch of polyglutamine residues, translated into a polyglutamine (polyQ) tract. The result is the formation of a mutantform of the *HTT*, which can be found in the brains of HD patients and animal models of HD.

Over the years, a number of different models for HD have been introduced varying from insects (*drosophilae melanogaster*) [11], invertebrate models as flatworms (*C. elegans*) [12], various rodent models, a transgenic ovine [13, 14], and pig model [15] to a recently developed nonhuman primate transgenic model [16]. This number of models is still limited, when compared to other neurodegenerative disorders such as Alzheimer's disease, for which currently hundreds of models exist. One explanation could be the low incidence of HD, and therefore it might not receive high priority by society and science. On the other hand, it is one of the few neurodegenerative diseases where the origin (i.e., mutation) has been so clearly described and thus where potentially a definite solution can be found.

Few years ago, the first transgenic rat model of HD (tgHD) was established by von Hörsten and coworkers [17]. The tgHD rats carry a truncated huntingtin cDNA fragment with 51 CAG repeats under control of the native rat *huntingtin* promoter and were reported to show motor and cognitive symptoms. In addition, a progressive striatal volume loss was documented [17]. These behavioural and neuropathological phenotypes, together with the larger size of the skull and brain in comparison to mice for therapeutic interventions, motivated us to work with this animal model. We have been using this animal model in our HD research and will review here our experience with relevant literature.

## 2. Behavioural Phenotype

HD is well known for its motor symptoms such as chorea and bradykinesia. However, the nonmotor symptoms of HD patients are also important. Huntington (1872) already reported nonmotor symptoms, such as insanity, depression, and poor impulse control [18]. An important question is whether these nonmotor symptoms are linked to the motor symptoms of HD and whether they share a common underlying mechanism, such as a hyperdopaminergic status [10] or striatal cell damage [9]. From a clinical perspective, there is evidence that nonmotor symptoms are mainly linked to hypokinesia and not so much to hyperkinesia (i.e., chorea) [19, 20]. In this respect, it is an ongoing debate whether hypokinetic and hyperkinetic symptoms in HD have a common neuronal substrate. Different transgenic animal models have been developed to characterise the motor and nonmotor symptoms in more detail. The widely used R6/2 mouse model of disease has been found to show less anxiety-related behaviour and exhibits hypokinetic features [21], and in the tgHD rats nonmotor symptoms and hyperkinetic features were found mimicking clinical HD [17]. In the latter model, already at 3 months of age, the animals showed signs of reduced anxiety in the social interaction test, while there was no evidence of motor or cognitive dysfunction [22]. This is in comparison to human data illustrating the presence of psychiatric symptoms, such as anxiety, prior to the onset of the motor symptoms, in the early stages of the disease. Though changes in anxiety in the transgenic rats show a reduction, patients often show increased anxiety levels, not related to disease stages. This might be partly explained by the fact that increased anxiety in patients often occurs due to uncertainty about the start and/or course of the disease. This phenomenon does not exist in animals.

In one of our studies, we investigated the relationship between motor and nonmotor behaviour in the earlier stages of the disease in the tgHD rat model by using motor and nonmotor tests [23]. We found tgHD animals to be hyperkinetic (increased distance moved) in the open field test compared to their wildtype littermates at all ages tested (6, 7, 8, and 10 months of age), which was accompanied by reduced anxiety-like behaviour in the open field test and the elevated zero maze, but not in the home cage emergence test. No major changes were found in hedonia (sucrose intake test) and motivation for food (food intake test). Our data suggest that hyperkinetic features and reduced anxiety in the tgHD rats are associated behaviours and are seen in the earlier stages of the disease. These data support the hypothesis that the neuronal substrate of the hyperactivity and reduced anxiety might be similar.

In a next study, we investigated the visual object and visuospatial memory capabilities of the tgHD rats by means of the object recognition task (ORT) and the object location task (OLT), respectively [24]. Visual object and visuospatial cognition are linked to the function of the basal ganglia, the primary site of neuropathology in HD. Subsequently, memory deficits are frequently seen in HD patients and are linked to striatal, especially caudate nucleus (dorsal striatum), and hippocampal alterations [25–27].

The ORT allows the assessment of object memory and is reviewed to be specific for assessing the functionality of the ventral memory pathway including the perirhinal cortex in rats [28]. The OLT assesses the spatial component of object memory determined by the dorsal memory pathway, which gives an estimation of the functionality of the parahippocampal cortex or its rodent equivalent, the postrhinal cortex [29]. Together these brain areas reflect the medial temporal lobe memory system, which has been considered as the declarative memory system [30]. Therefore, we tested the subjects at two different time points, 10 and 16 months, corresponding to early and late stages of the HD. We found memory deficits in the tgHD rat model, for both visual object and visuospatial memory, at the early and late stages of the disease. These rodent data are comparable to data of human studies demonstrating impairment of both visual object and visuospatial memory too. Deficits in striatal cell activity, hippocampal long term potentiation (LTP) functionality, and decreased levels of Brain derived neurotrophic factor (BDNF) have been associated with memory deficits in HD patients and transgenic mouse models of HD [31–34]. Nonetheless, further studies are needed to clarify these mechanisms and their implications in the tgHD rat model. In this respect, a recent study showed the presence of prefrontostriatal processing alterations using a behavioural and electrophysiological paradigm, which could also underlie some of the cognitive symptoms seen in this model [35].

In another study, we further extended the behavioural profile of this model in the later stages of the disease. Specifically, we tested the hypothesis that these rats exhibit age- and genotype-dependent changes in cognitive performance and chorea-like symptoms (abrupt, rapid, brief, and unsustained irregular movements of the neck) [36]. Rats were evaluated in a choice reaction time task to study the cognitive performance, including reaction time and premature responding, and in an open field setting to rate the choreiform movements. We found that tgHD rats showed a clear progression of cognitive and motor impairment over time. At 15 months of age, the homozygous (+/+) tgHD rats showed only a slight impairment in the number of premature responses, whereas at 20 months of age, this impairment was significantly increased, and more cognitive deficits such as a decreased number of correct responses became apparent compared to hemizygous (+/−) and wildtype (−/−) littermates. Furthermore, the number of choreiform movements in the homozygous tgHD rats increased significantly from 15 months to 20 months of age, in comparison to hemizygous and wildtype littermates. Both cognitive and motor impairments were significantly more pronounced in the homozygous tgHD rats as compared to the hemizygous tgHD rats, which is indicative of a gene-dose effect.

## 3. Neurophysiological Phenotype

The tgHD rats show a specific behavioural phenotype, consisting of hyperkinetic movements, impaired anxiety and memory parameters, and impulsivity, at different stages of the disease. One of questions arising is whether these behavioural symptoms have neurophysiological substrates.

A link between symptoms and neurophysiological substrate has been well established in another movement disorder, Parkinson's disease (PD). In PD, which is histopathologically characterized by selective, chronic, and progressive nigrostriatal degeneration, the subthalamic nucleus (STN) displays a continuous abnormal “bursting” mode of activity whereas in physiological conditions it exhibits a more or less regular pattern of discharge with intervals of burst activity [37–39]. This so-called STN hyperactivity is held responsible for at least part of the cardinal PD symptoms such as hypokinesia/bradykinesia and rigidity [40–42]. Interestingly, the discovery of STN hyperactivity has been the result of systematic scientific research. In short, in 1987 Miller and DeLong demonstrated that the STN exhibited increased neuronal activity in MPTP-treated primates, in an electrophysiological study exploring the activities of basal ganglia nuclei [43]. These observations were the basis for the pioneering work of Bergman and colleagues [44]. They showed that lesions of the STN reduced all of the major motor disturbances in monkeys rendered parkinsonian by MPTP. Benabid and coworkers introduced deep brain stimulation (DBS) as an alternative for ablative surgery in movement disorders [45] and were the first, motivated by the results of the lesion studies and by a crucial stimulation study in primates in the same year [46], to explore the effects of STN DBS in a patient suffering from advanced PD [47]. In 1995, the results of 3 patients were published showing that bilateral STN DBS resulted in marked improvement in motor symptoms [48]. This report was actually the beginning of the successful STN DBS era.

**Figure 1:**
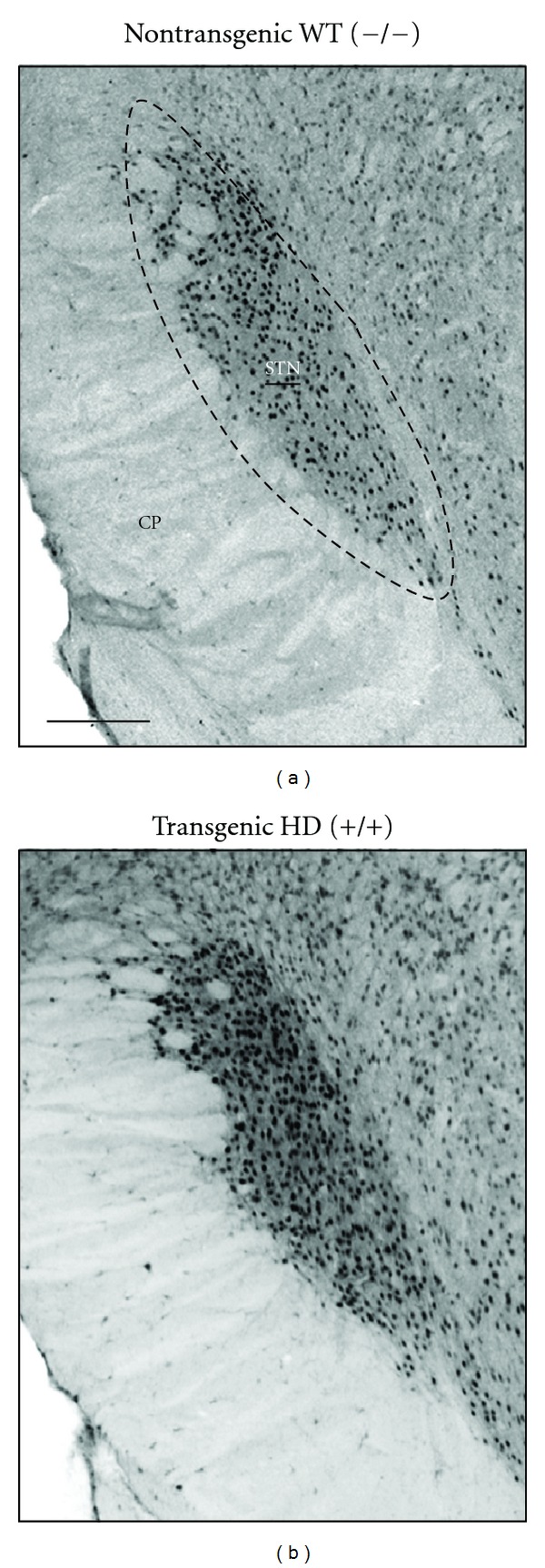
Representative low-power photomicrographs of frontal brain sections stained for PGC-1*α* showing the subthalamic nucleus (STN) of a control and a transgenic HD rat. Note the increased number of PGC-1*α*-containing cells STN of the tgHD rat upon close inspection in comparison with the control subject. Scale bar is approximately 250 *μ*m. The anatomical level is approximately anteroposterior −4.16 mm from bregma according to the rat brain atlas of Paxinos and Watson of 1998. Adopted from Vlamings et al. [8].

In line with the advances in PD, we aimed to investigate the metabolic and electrophysiological activities of the basal ganglia nuclei, consisting of the striatum, GP (globus pallidus, the homologue of globus pallidus externus in primates), EP (entopeduncular nucleus, rodent equivalent of the primate globus pallidus internus), substantia nigra pars compacta (SNc), and reticulata (SNr), and the STN in the tgHD rats [8]. We reasoned that mapping of neuronal and metabolic activity of the basal ganglia nuclei would help in identifying a functional substrate for symptoms and therapeutic interventions in HD. To determine the *overall* neuronal activity of all cells (supracellular) per basal ganglia nuclei, a cytochrome oxidase (COX) enzymatic staining was performed, and optical densities were measured at the level of each basal ganglia nuclei. Secondly, we performed *single-unit* electrophysiological recordings, which reflect the activity of the basal ganglia nuclei at the cellular level. Finally, to investigate the subcellular activity of the various basal ganglia nuclei we performed an immunohistochemical staining for peroxisome proliferator-activated receptor-*γ* coactivator (PGC)-1*α*, a transcription coactivator, and a key player in the mitochondrial energy apparatus. PGC-1*α* induces and coordinates gene expression that stimulates mitochondrial oxidative metabolism in various tissues. It is highly expressed in tissues with increased energy demands and large numbers of mitochondria [49]. In tgHD rats, optical density analysis showed a significantly increased cytochrome oxidase levels in the GP and STN when compared to controls. PGC-1*α* expression was only enhanced in the STN (Figure 1), and electrophysiological recordings revealed increased firing frequency of the majority of the neurons in the STN and a reduced firing frequency in the GP [8].

The finding that the STN shows enhanced activity at different functional levels cannot be explained based on the classical theories on the corticobasal ganglia-thalamocortical circuits in HD [50]. Namely, the expectation would be decreased or impaired activity of the STN and an elevated GP activity due to a loss of striatal medium spiny neurons expressing D2 receptors and enkephalin. Here, we found an increase in the COX activity in the GP in the tgHD animals, which is in line with the previously mentioned theory, but we failed to find support from the PGC-1*α* expression and electrophysiological studies. It might well be that subtle changes in activity can be detected with COX histochemistry, which represents overall activity (sum of inhibited, excited, and unchanged cell activities). The changes in the STN were more robust and present at subcellular, cellular, and supracellular levels. We think that the explanation for this change is through the direct dopaminergic input from the SN pars compacta [51]. In the tgHD rats, we found elevated levels of tyrosine hydroxylase (TH), rate-limiting enzyme in the synthesis of dopamine, in the striatum due to increased number of dopamine-containing cells in the SN pars compacta [10]. It has been shown that higher concentrations of dopamine have a dual effect on STN neurons, increasing the firing rate and changing the pattern of firing into a more regular mode [52]. This is what we have observed here. The elevated levels of dopamine in HD seem to induce more neurobiological changes in the basal ganglia than previously expected. Another contributing factor to enhanced STN activity could be the reduced activity of the regular neurons of the GP. Altogether, our results suggested that the STN and GP play a role in the symptoms of HD and can be a potential target for therapeutic interventions.

## 4. Neuropathological Phenotype

The neuropathology of HD includes profound and progressive neuron death in the striatum and, to a lesser extent, in the cortex [53]. Several animal models represent some of these features of HD. For instance, injection of excitotoxins and mitochondrial toxins can mimic some aspects of the neuropathology of HD, but the resulting neuronal death is not progressive. Genetically modified mouse models have further helped to understand the pathogenesis and molecular mechanisms of the illness [54]. Major drawbacks of these models, however, are the rapid disease progression (as in the case of the most commonly used model, namely, the R6/2 mice) and the relatively small size of the mouse brain, limiting the usefulness of these mouse models for longitudinal studies and for therapeutic approaches based on surgical intervention. Except for studies reporting striatal neuron loss in the YAC128 mouse model [55], substantial striatal neuron loss has not really been observed in transgenic mouse models of HD.

**Figure 2:**
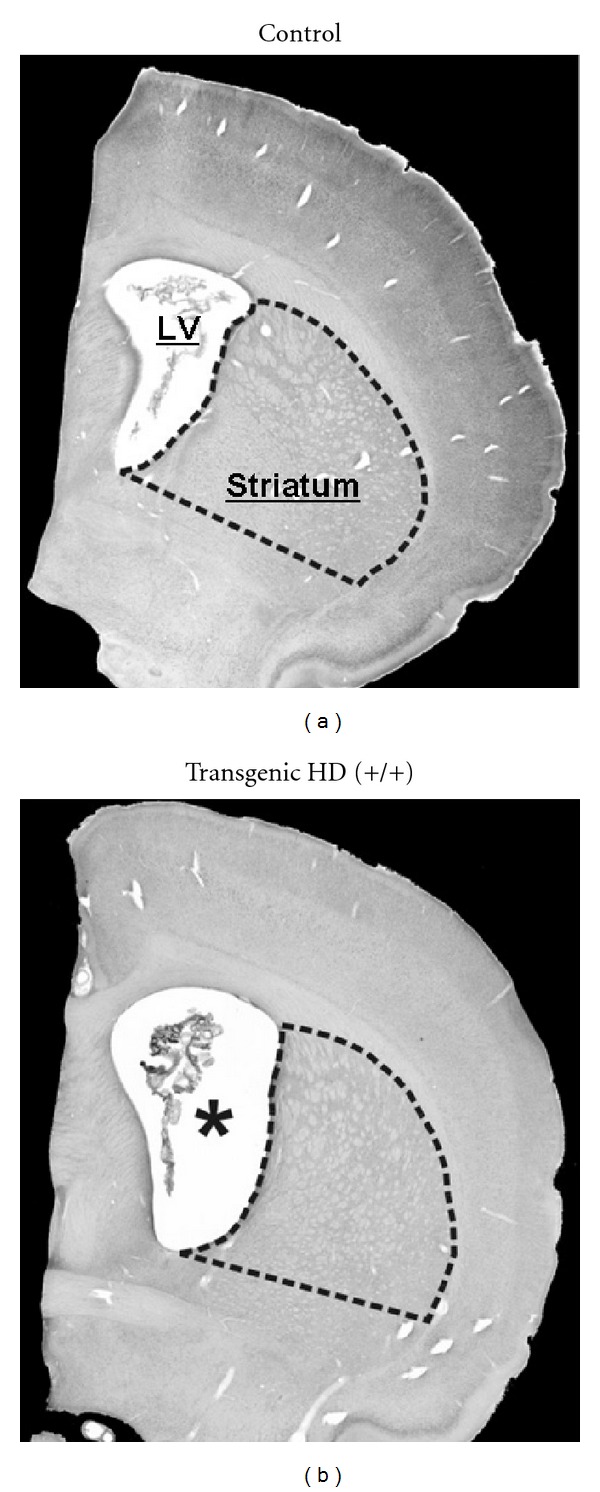
Representative photomicrographs of Nissl-stained frontal sections of the brains from a 12-month-old control rat and HD transgenic rat. Note the smaller striatum and the larger lateral ventricle (LV) (asterisk in b). The anatomical level is approximately anteroposterior 1.60 mm from bregma according to the rat brain atlas of Paxinos and Watson of 1998. Adopted from Kantor et al. [9].

In the first publication on the tgHD rats, striatal shrinkage and enlarged lateral brain ventricles in magnetic resonance images at 8 months of age were reported [17]. In a later study, the striatal neuron loss during the disease course and aggregates were documented [22]. We further characterized these changes in a histopathological study [9]. We found a reduction in striatal volume in the brains of 12-month-old tgHD rats compared with wildtype littermates (Figure 2). This age-related volume reduction was more pronounced in the medial, paraventricular part of the striatum, corresponding to the regions where the earliest neuropathological changes are seen in human HD [56]. Moreover, high-precision, design-based stereological analysis showed statistically significant neuron loss in striatum but not in frontal cortical layer V at 12 months of age. Thorough microscopical examination of Nissl stained sections revealed no neurons with abnormal morphology in the striatum of tgHD rats and no signs of astroglia activation. Nevertheless, dysregulation of neuronal firing patterns of striatal cells was found in these animals [57]. Of particular importance was the finding of dark, pycnotic pyramidal cells mostly in layer V of the frontal cortex of tgHD rats, resembling pycnotic pyramidal cells seen in the motor cortex of human HD patients [58]. Furthermore, enhanced accumulation of autofluorescent material (most probably representing lipofuscin) was specifically observed in layer V pyramidal cells of tgHD rats, resembling enhanced lipofuscin accumulation in the human disease. These findings indicate chronic neurodegenerative processes in the motor cortex of tgHD rats.

Another line of investigation is the pathoanatomical basis for HD chorea. A link with the dopaminergic system has been suggested by human postmortem studies and clinical therapy studies. Early postmortem studies showed that striatal dopamine levels, both in the dorsal and ventral striatum, were significantly higher in HD patients compared to controls [59–61]. In addition, clinical studies have shown that the chorea can be treated with dopamine antagonist or dopamine-depleting drugs [62].

**Figure 3:**
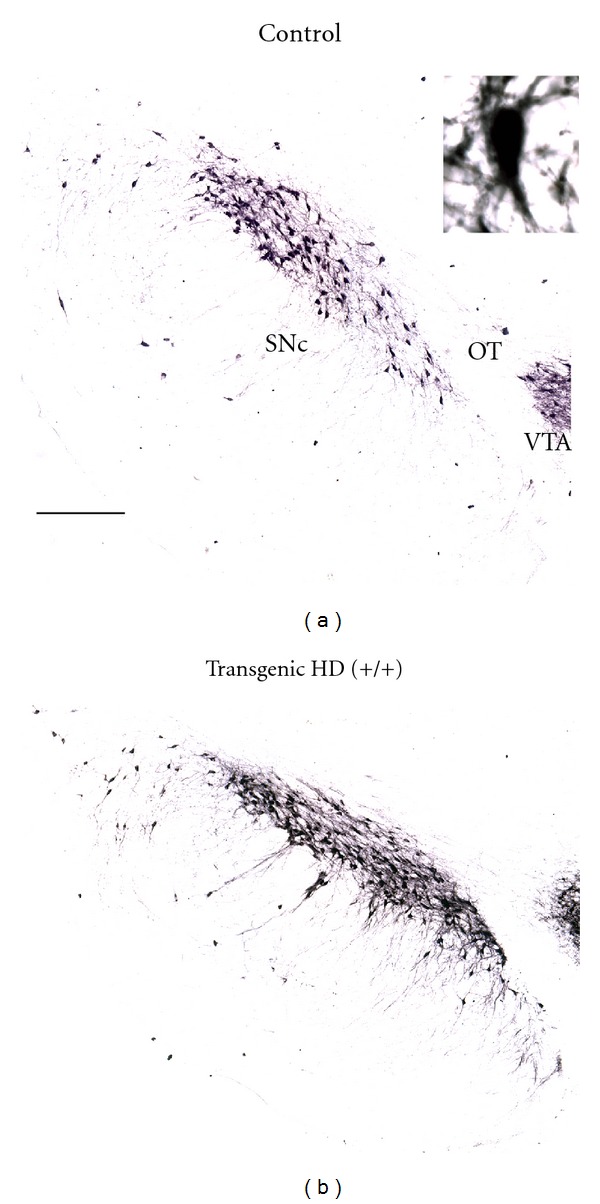
Representative low-power photomicrographs of frontal brain sections stained for tyrosine hydroxylase (TH) showing the substantia nigra pars compacta (SNc), optic tract (OT), and a small part of the ventral tegmental area (VTA) of a control and a transgenic HD rat. Note the increased TH-containing cell density in the SNc of the transgenic HD rats upon close inspection. The high-power photomicrograph inset in the right upper corner shows a magnification of TH-containing cells of a transgenic rat. Scale bar is approximately 250 *μ*m. The anatomical level is approximately anteroposterior −5.2 mm from Bregma according to the Paxinos and Watson atlas of 1998. Adopted from Jahanshahi et al. [10].

The origin of elevated dopamine levels in the dorsal and ventral striatum in HD remains unknown. In another histopathological study [10], we tested the hypothesis that elevated striatal dopamine levels are caused by changes in the SNc and the ventral tegmental area (VTA), since these regions are the main source of striatal dopamine [63, 64]. We used antibodies raised against TH, the rate-limiting enzyme in the synthesis of dopamine, and by means of stereological counting methods analysed the number of TH-containing cells in the SNc and VTA, in the only experimental model of HD with chorea, the tgHD rats. We found increased expression of TH in the striatum of tgHD rats. This reflects increased striatal dopamine levels, since TH enzymatic activity highly corresponds to the cellular levels of dopamine [65]. Stereological counts of TH-containing cells revealed a substantial increase in the number of TH containing cells in both regions in tgHD rats when compared to controls (Figure 3). Our findings suggest that the origin of the increased dopamine in the striatum comes from the two main nuclei supplying the striatum with dopamine, the SNc for the nigrostriatal dopamine pathway and the VTA for the mesolimbic dopamine pathway. Since it has been demonstrated that the total number of cells in the substantia nigra in postmortem HD brains was not different from age-matched controls [66], it is possible that the increased number of TH containing cells in the SNc and VTA is the result of a change in phenotype of the non-TH-containing cells. For the SNc it is known that about 45% of the cells are non-TH containing [67]. A change in the phenotype of a TH cell, while the cell is still functional, has been documented before in the substantia nigra of mice [68] and rats [69].

## 5. Conclusion

We have been using the tgHD rats for several years in our research. We have experienced that these rats have a slow progressive behavioural and neuropathological phenotype. In general, we found out that the symptoms can be divided in two to three stages: early, (middle), and late. The early stage is characterised by hypermobility and reduced anxiety behaviour [23]. Although some scientists have found subtle cognitive declines [22, 35], in our hands these do not have a large impact on the animals. We have not found choreiform movements, at this early disease stage. This stage is not accompanied with striatal cell degeneration or cortical cell damage, which occurs later in the disease [9, 22]. Nevertheless, there might already be striatal cell dysfunction [57] which continues in the middle phase (>8–10 months of age). Appearance of the first choreiform movements starts during this disease stage with an increase in the last disease stages (>15–24 months of age). This later stage is mainly characterised by the presence of more choreiform movements at the level of the head, neck, and limbs. Upon testing, the animals show impaired cognitive functioning and impulsivity-like features [24, 36]. There is a profound loss of striatal cells and signs of cortical cell damage [9]. The life expectancy of these animals is shorter than controls, probably a few months (unpublished observations).

There are some issues, which need to be considered in this animal model. The first is the effect of sex. There are differences in the behavioural phenotype between the sexes [70]. This needs to be taken into account when comparing sets of data. Therefore, in our studies, we use animals of only one sex male or female per experiment. In addition, we prefer to use merely male animals, like in most behavioural studies, to exclude any possible effects of the oestric cycle in female animals. The second is the gene-dose effect. We have been working with both homozygous (+/+) and hemizygous (+/−) transgenic animals in a few studies [10, 36]. Homozygous animals show more robust behavioural and neuropathological features [9, 36], mimicking human HD. Therefore, we decided to work with homozygous rats, only. The third issue is a potential gene-drift effect. In a recent publication, researchers could not establish robust cognitive changes in this animal model [71, 72]. One explanation could be a potential gene drift, but the existence of such mechanism still needs to be demonstrated. In our colony of animals, we have observed slight differences between homozygous rats but consistently found clear behavioural and neuropathological phenotypes.

After evaluating the different phenotypes of the tgHD rats, we consider this animal model suitable to evaluate therapeutic approaches.
